# Lipocalin-2 in neutrophils induces ferroptosis in septic cardiac dysfunction *via* increasing labile iron pool of cardiomyocytes

**DOI:** 10.3389/fcvm.2022.922534

**Published:** 2022-08-04

**Authors:** Yuxue Huang, Ning Zhang, Cuiping Xie, Yayu You, Lei Guo, Feiming Ye, Xiaojie Xie, Jian’an Wang

**Affiliations:** Department of Cardiology, Cardiovascular Key Laboratory of Zhejiang Province, Second Affiliated Hospital, Zhejiang University School of Medicine, Hangzhou, China

**Keywords:** lipocalin-2, ferroptosis, labile iron pool, septic cardiac dysfunction, neutrophil, neutrophil gelatinase-associated lipocalin

## Abstract

Cardiac dysfunction is a common complication of sepsis with high mortality. The present study was designed to identify the effect of neutrophil-derived lipocalin-2 (LCN2) in septic cardiac dysfunction (SCD) and its potential mechanism. Wild-type (WT) and LCN2-knockout (LCN2 KO) mice were peritoneally injected with lipopolysaccharide (LPS) to induce SCD. The cardiac function was assessed 12 h after LPS injection by echocardiography. Cardiac tissue was harvested for the evaluation of malonaldehyde (MDA) and prostaglandin E synthase 2 (PTGS2) mRNA levels. LPS induced ferroptosis and SCD in mice. LCN2 deficiency attenuated cardiac injury post-LPS administration. *In vitro*, LCN2 expression in neutrophils increased in response to LPS. Ferroptosis of cardiomyocytes induced by conditioned medium (CM) from LPS-induced neutrophils of WT mice could be attenuated in CM from LPS-induced neutrophils of LCN2 KO mice. Exogenous LCN2 induced H9C2 cell ferroptosis *via* increasing labile iron pool (LIP). In conclusion, our results showed that LCN2 deficiency prevented heart dysfunction and ferroptosis in SCD mice and suggested that neutrophil-derived LCN2 might be a promising therapeutic target for SCD.

## Introduction

Sepsis is identified as a systemic dysregulated immune response to infection or injury, causing life-threatening multiple organ dysfunction. Cardiac dysfunction is present in 10–70% of septic patients and may lead to high mortality as 70–90% in subjects with sepsis (1, 2). Neutrophils, as the most abundant innate immune cells, can immigrate to infected tissues, and exert anti-infection roles, including phagocytosis, degranulation, and formation of neutrophil extracellular traps (NETs) (3). It has been demonstrated that neutrophil recruitment is important for lipopolysaccharide (LPS)-induced septic mouse models (4).

Lipocalin-2 (LCN2), also known as neutrophil gelatinase-associated lipocalin (NGAL), siderocalin, or 24p3, belongs to the lipocalin superfamily. It is a β-barrel-secreted protein that binds to siderophore-complexed ferric iron with high affinity, which involves various inflammatory processes (5, 6). LCN2 was reported as a biomarker of kidney injury (7) and increased in the neutrophils of patients with alcoholic steatohepatitis (8). High plasma LCN2 also predicted high mortality and cardiac dysfunction in severe sepsis and septic shock patients (9). In the GALLANT trial, plasma LCN2, along with B-type natriuretic peptide, is deemed as a biomarker for patients with acutely decompensated heart failure (10). As for patients with chronic heart failure, plasma LCN2 serves as a predictor for clinical outcomes, including mortality and prognosis (11, 12). The divergent biological roles of LCN2 can be mediated by three receptors on cell membranes, namely, 24p3R, LRP2, and MC4R. 24p3R is the major receptor widely expressed in various cell types of humans and mouse models (6). The binding complex of LCN2/24p3R can carry iron and be internalized into the cytoplasm and subsequently increase intracellular iron (13). It is still unknown whether LCN2 is involved in LPS-induced septic cardiac dysfunction (SCD).

Ferroptosis is a newly identified form of regulated cell death discovered by Dixon et al. with a distinctive feature of iron-dependent lipid peroxidation (14–16). The fact that iron is a key factor in ferroptosis is supported by various experimental studies on the ability of iron chelators to attenuate ferroptosis (14, 15, 17, 18). Cellular labile iron pool (LIP), a transitory pool of chelatable and redox-active iron, serves as the crossroad of cell iron metabolism (19). Growing evidence has shown that ferroptosis is critical for many cardiovascular diseases, such as doxorubicin-induced cardiomyopathy and ischemia/reperfusion injury (20, 21). There are some reports of LPS in triggering myocardial pyroptosis, apoptosis (22), autophagy (23), necroptosis (24), and ferroptosis (25).

In this study, we established an LPS-induced SCD mouse model and found that LCN2 expression significantly increased in cardiac tissue, which was dominantly derived from neutrophils. Exogenous LCN2 led to myocardial ferroptosis by increasing free iron of LIP *in vitro*. *In vivo*, LCN2 deficiency improved the cardiac function of mice in response to LPS. Our results suggested that neutrophil-derived LCN2 might be a promising therapeutic target for SCD.

## Materials and methods

### Mice

Wild-type (WT) and LCN2 knockout (LCN2 KO) mice with a C57BL/6J background were purchased from SLAC Laboratory (Shanghai, China) and GemPharmatech (Jiangsu, China), respectively. The mice were bred in-house under specific pathogen-free conditions with free access to a normal chow diet and water, at a constant temperature (22 ± 2^°^C) and humidity (60–65%) with a 12-h dark/light cycle. All studies were performed in compliance with guidelines of the Institutional Animal Care and Use Committee at Second Affiliated Hospital, Zhejiang University School of Medicine.

### *In vivo* drug administration

The genotypes were verified by PCR using primer sets for LCN2 KO and WT. In the study, 6–8-week-old male mice were used. Lipopolysaccharide (LPS) was purchased from Sigma-Aldrich (25 mg/kg, Escherichia coli 0111: B4) and dissolved in sterile phosphate-buffered saline (PBS), as described previously (26). Either LPS or PBS was injected intraperitoneally for 12 h.

To investigate the role of ferroptosis in the LPS-induced mice, WT male mice were intraperitoneally injected with Fer-1 (HY-100579/CS-0019733, MCE, United States) at a dose of 1 mg/kg per mouse or vehicle 24 and 2 h prior to LPS administration (21). Fer-1 was dissolved in DMSO and then diluted 1:9 in corn oil. The mice were inspected every 4 h for 3 days.

At termination, the right atrium was cut open, and PBS was perfused through the left ventricle to remove blood in the aorta. Subsequently, serum and cardiac tissue were collected and stored at –80^°^C for protein and RNA analyses.

### Echocardiography and hemodynamics

Echocardiography was performed using a Vevo 2100 system (VisualSonics, Toronto, Canada). Two-dimensional and M-mode echocardiographic images at the papillary muscle level were obtained by two investigators independently. Left ventricle internal diameters at end-diastole and end-systole (LVIDd and LVIDs, respectively) were measured. The LV ejection fraction (EF), fractional shortening (FS), left ventricular end-diastolic volume (LVEDV), and left ventricular end-systolic volume (LVESV) were analyzed independently by three investigators who were blinded to the experimental design (27).

The closed-chest approach was used to obtain the LV hemodynamics in anesthetized mice using a Millar MPVS-300 system equipped with a Millar SPR-839 catheter, according to described previously (28). Briefly, the anterior thorax and the neck of the mouse were shaved upon complete anesthesia. The neck of the mouse was then opened with a sagittal incision to expose the trachea. The conductance micromanometer was inserted into the right common carotid, and the catheter was placed in the left ventricle under the guidance of online pressure signal. The following indicators were used for the continuous monitoring of hemodynamics, left ventricular end-systolic pressure (LVSP), left ventricular end-diastolic pressure (LVEDP), and maximal left ventricular pressure rising and decreasing rates (LV ± dp/dt max). Data were analyzed using Lab Chart Pro software (AD Instruments).

### *In vitro* cell culture

H9C2 cell line and RAW 264.7 cell line were purchased from the Cell Bank of Shanghai Institutes for Biological Sciences, Chinese Academy of Sciences. The H9C2 cells were cultured in Dulbecco’s modified Eagle medium (DMEM, high glucose, Gibco) supplemented with 10% fetal bovine serum (FBS, Hyclone), as described previously (29). Cardiomyocytes and cardiac fibroblasts were isolated from the ventricles of neonatal mice by using the Neonatal Cardiomyocyte Isolation Kit (Cat#130100825, Miltenyi BioTec, Teterow, Germany) according to manufacturer’s instructions and cultured in DMEM supplemented with 10% FBS, as described previously (30). Neutrophils were isolated from peripheral blood by using a Mouse Neutrophil Isolation kit (Cat#LZS1100, TBD, China) according to the manufacturer’s instructions. RAW 264.7 cells and neutrophils were cultured in RPMI-1640 medium (Gibco) supplemented with 10% FBS, as described previously (31, 32).

The H9C2 cells were incubated with recombinant murine LCN2 (rmLCN2, Cat#CM17; Novoprotein, Shanghai, China) for 24 h at a final concentration of 1 μg/ml. Pre-incubation with either ferrostatin-1 (Fer-1, 10 μM) or deferoxamine (DFO, 25 μM) was carried out in H9C2 cells for 2 h prior to incubation with rmLCN2 at a final concentration of 1 μg/ml, and the cells were collected for next experiments.

Neutrophils and RAW 264.7 cells were incubated with either RPMI-1640 or LPS for 12 h at a final concentration of 100 ng/ml or vehicle. Neonatal cardiomyocytes and cardiac fibroblasts were incubated with either LPS for 24 h at a final concentration of 1 ug/ml or vehicle. The conditioned medium (CM) of neutrophils was harvested to incubate neonatal cardiomyocytes for another 24 h, and the cells were collected for next experiments.

### Cell transfections

The H9C2 cells were transfected with 100 nM of 24p3R siRNA and its non-specific siRNA negative control (RiboBio, Guangzhou, China), using Lipofectamine 3000 (Cat#13778030, Invitrogen). Quantitative PCR was used to determine 24p3R expression in the transfected cells after 48 h.

### Flow-cytometric analysis

Single cell suspension was prepared and stained at 4°C in PBS with antibodies against CD45 (CAT561869, BioLegend, Beijing, China), CD11b (CAT101212, BioLegend, Beijing, China), Ly6G (CAT127605, BioLegend, Beijing, China), and F4/80 (CAT123109, BioLegend, Beijing, China) for 30 min. Flow cytometric assay was performed using a CytoFLEX FACS machine (Beckman Coulter, Brea, CA), and data were analyzed by FlowJo (FLOWJO, LLC, Ashland, OR).

### Enzyme-linked immunosorbent assay and western blotting

LCN2 levels of serum and tissue lysates were measured using enzyme-linked immunosorbent assay (ELISA) kits (Cat# DY1857-05, DY008, R&D, United States) according to manufacturer’s recommendation.

Protein lysate samples were prepared from cells or snap-frozen tissues in RIPA solution (Cat# P0013B; Beyotime) supplemented with protease inhibitor (Cat# 05892791001; Roche). Denatured protein lysates were resolved by 10 and 12% (wt/vol) SDS-PAGE gels. After transfer, membranes were incubated with primary antibodies against GPx4 (1:1000,Cat# ET1706-45, HUABIO, Hangzhou, China), FSP1 (1:1000,Cat# ER62655, HUABIO, Hangzhou, China), and β-actin (1:3,000,Cat# KC-5A08, Kangcheng, Sichuan, China), LCN2 (1:1,000,Cat# ab216462, abcam, United States) overnight at 4^°^C and subsequently incubated with horseradish peroxidase (HRP)-conjugated secondary antibodies, which were detected by enhanced chemiluminescence (Cat# WBKLS0500; Millipore). Immunoblots were analyzed using Image Lab software (Bio-Rad).

### Quantitative real-time PCR

Total RNA was isolated from cells or snap-frozen tissues using TRIzol (Takara), and RNA concentration and purity were measured by spectrophotometry. RNA was reverse-transcribed using the PrimeScript RT reagent Kit (Takara) in accordance with the manufacturer’s instructions. Quantitative PCR was performed using a CFX96 Real-Time System (Bio-Rad) and SYBR Green Supermix (Bio-Rad) in accordance with the manufacturer’s instructions. The fold difference in gene expression was calculated using the 2^–△△Ct^ method and is presented relative to *actin* mRNA. All reactions were performed in triplicate, and specificity was monitored using melting curve analysis. See Table 1 for the PCR primers used.

**TABLE 1:** Primers for qPCR (sequence: 5′- > 3′).

PTGS2 forward primer	TTCAACACACTCTATCACTGGC
PTGS2 reverse primer	AGAAGCGTTTGCGGTACTCAT
LCN2 forward primer	TGGCCCTGAGTGTCATGTG
LCN2 reverse primer	CTCTTGTAGCTCATAGATGGTGC
SLC7a11 forward primer	GGCACCGTCATCGGATCAG
SLC7a11 reverse primer	CTCCACAGGCAGACCAGAAAA
ACSL4 forward primer	CTCACCATTATATTGCTGCCTGT
ACSL4 reverse primer	TCTCTTTGCCATAGCGTTTTTCT
ALOX12 forward primer	TCCCTCAACCTAGTGCGTTTG
ALOX12 reverse primer	GTTGCAGCTCCAGTTTCGC
ALOX15 forward primer	GGCTCCAACAACGAGGTCTAC
ALOX15 reverse primer	AGGTATTCTGACACATCCACCTT
IL-1 β forward primer	GTGCTACTGGGGCTCATTTGT
IL-1 β reverse primer	GGAGTAAGAGGACACTTGCGAAT
TNF α forward primer	CCCTCACACTCAGATCATCTTCT
TNF α reverse primer	GCTACGACGTGGGCTACAG
IL-6 forward primer	TAGTCCTTCCTACCCCAATTTCC
IL-6 reverse primer	TTGGTCCTTAGCCACTCCTTC
Actin forward primer	GGCTGTATTCCCCTCCATCG
Actin reverse primer	CCAGTTGGTAACAATGCCATGT

### Lipid peroxidation assay

Lipid peroxidation was measured by malondialdehyde (MDA) and live cell analysis reagent BODIPY 581/591 C11 (redox-sensitive dye). The MDA level (Cat#A003-1-2, Jiancheng, Jiangsu, China) was measured according to the manufacturer’s protocol. Briefly, lipid peroxide reacted with chromogenic reagents under the condition of 98^°^C for 40 min and produced a stable chromophore with a maximum absorption peak at 532 nm. For BODIPY 581/591 C11 (Cat#D3861, Invitrogen, United States) staining, the cells were incubated in the dark with the reagent at a working concentration of 2.5 μM at 37^°^C for 30 min, and then the cells were stained with Hoechst nuclear stain (Beyotime,C1017,China) for 10 min. Subsequently, the cells were washed with PBS. Images were acquired under an IX83 fluorescence microscope (Olympus, Tokyo, Japan). Analysis of BODIPY 581/591 C11 was performed by measuring the intensity of fluorescence. Images were from at least six randomly selected regions of interest across three independent experiments. The fluorescence intensity was measured by ImageJ (National Institutes of Health).

### Labile iron pool measurements

Labile iron pool was measured by calcein AM, as reported previously (33). Cells at a density of 2 × 10^5^ per well were incubated with calcein AM (Cat#C2012, Beyotime, Shanghai, China) at a finial concentration of 0.05 μM for 30 min prior to incubation with DMEM for another 30 min and rinsing with PBS. Flow cytometric assay was performed using a CytoFLEX FACS machine (Beckman Coulter, Brea, CA) at a 488-nm laser on an FL1 detector.

### Statistical analysis

GraphPad Prism version 7.0 (GraphPad Software) was used for statistical analyses. To compare continuous response variables between two groups, an unpaired two-tailed Student’s *t*-test was applied for normally distributed variables that passed the equal variance test, and the Mann–Whitney *U*-test was used for variables not passing either the normality or equal variance test. To compare more than two groups, one-way ANOVA was performed for normally distributed variables that passed the equal variance test and Kruskal–Wallis one-way ANOVA on ranks using the Dunn method for variables failed to pass the normality or equal variance test, respectively. *P* < 0.05 was considered statistically significant. Data that passed the equal variance test were represented as mean ± SEM. Data that did not pass either the normality or equal variance test were shown as median with 95% CI.

## Results

### Neutrophil-derived lipocalin-2 was increased in lipopolysaccharide-induced septic cardiac dysfunction mice

**FIGURE 1:**
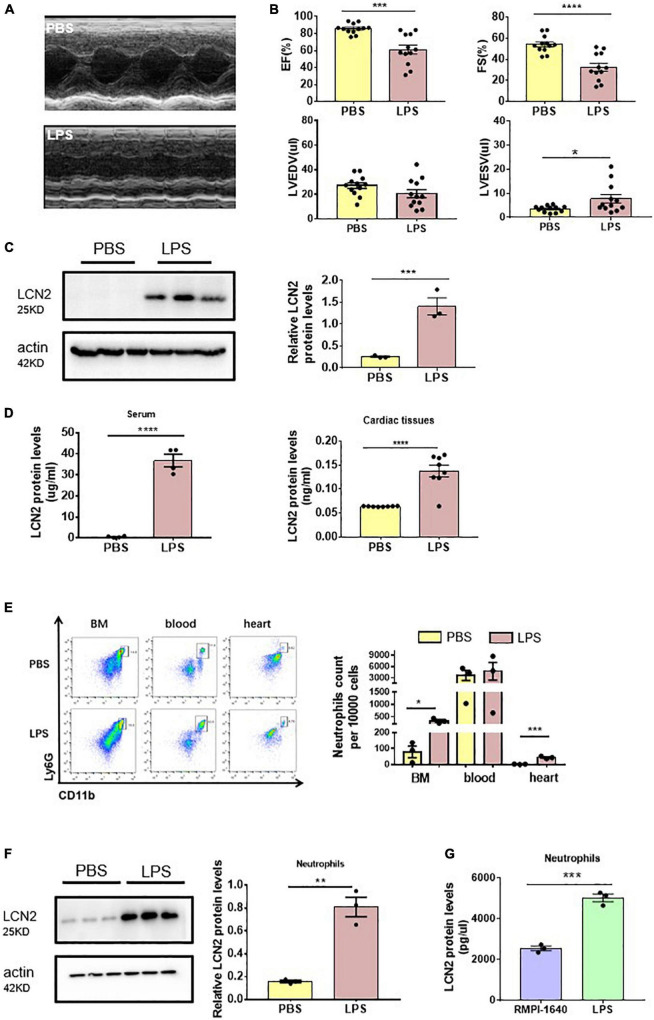
Neutrophil-derived LCN2 was increased in LPS-induced SCD mice. **(A,B)** Echocardiography was performed 12 h after intraperitoneal injection of either PBS or LPS (25 mg/kg) in WT mice and their representative images (*n* = 12 in each group). **(C)** Western blot was used to analyze LCN2 expressions in heart tissues 12 h after intraperitoneal injection with either PBS or LPS (*n* = 3 in each group). **(D)** ELISA was performed to detect serum (*n* = 4 in each group) and cardiac LCN2 protein levels (*n* = 8 in each group) 12 h after intraperitoneal injection with either PBS or LPS in WT mice. **(E)** Flow cytometric analysis was performed to determine the number of CD45+CD11b+Ly6G+ cells in the bone marrow (BM), blood, and heart 12 h after intraperitoneal injection with either PBS (*n* = 3) or LPS (25 mg/kg, *n* = 3) in WT mice. **(F)** Western blot was used to analyze the LCN2 expressions in neutrophils isolated from peripheral blood 12 h post-intraperitoneal injection of PBS or LPS (25 mg/kg, *n* = 6 in each group) in WT mice. **(G)** ELISA was performed to detect the LCN2 protein levels in neutrophils after 12-h incubation with either RMPI-1640 or LPS (100 ng/ml, *n* = 3 in each group). Measurement data are presented as mean ± SEM. *p < 0.05, **p < 0.01, ***p < 0.001, ****p < 0.0001.

To investigate the involvement of LCN2 on SCD, 6 to 8-week-old male C57Bl/6 mice were injected with either LPS (25 mg/kg) or PBS intraperitoneally for 12 h. Compared to the PBS group, EF and FS were significantly decreased (Figures 1A,B), indicating SCD establishment. We also found LVESV was increased in SCD mice, while LVEDV was not changed (Figures 1A,B). qPCR demonstrated that LCN2 mRNA was significantly upregulated in many organs, including the heart, liver, lung, and the kidney, but not spleen (Supplementary Figure 1A). Furthermore, compared to the PBS group, Western blot indicated that LCN2 expression in the myocardial tissues of LPS-induced SCD mice was significantly increased (Figure 1C). ELISA was performed to show that LCN2 protein concentrations elevated in the serum and myocardial lysates in LPS-induced SCD mice, compared to the PBS group (Figure 1D).

According to the previous studies that LCN2 was dominantly from neutrophils, we screened mononuclear cell suspension in myocardial lysates, peripheral blood, and bone marrow for LCN2 expression. As a result, neutrophils (as CD45 + CD11b + Ly6G + cells by flow cytometry) were obviously increased in response to LPS in both the bone marrow and cardiac tissues, and in peripheral blood, there was a tendency of increasing neutrophils (Figure 1E). LCN2 protein expression by Western blot was dramatically upregulated in neutrophils isolated from the peripheral blood of LPS-induced SCD mice, compared to the PBS group (Figure 1F).

To further verify the cell origin of LCN2, neutrophils, RAW 264.7 cells, neonatal cardiomyocytes, and cardiac fibroblasts were cultured *in vitro* and incubated with or without LPS for 12 h. ELISA detection indicated that LCN2 secreted by neutrophils was much more than that secreted by RAW 264.7 cells (Figure 1G and Supplementary Figure 2A). However, Western blot indicated that LCN2 expression was not upregulated in neonatal cardiomyocytes or cardiac fibroblasts in response to LPS (Supplementary Figure 2B). Taken together, these data suggested that LCN2 was involved in the pathogenesis of LPS-induced SCD, which was mainly derived from neutrophils.

### Lipocalin-2 deficiency improved cardiac function in lipopolysaccharide-induced mice

In order to investigate the roles of LCN2 in SCD, we purchased LCN2-knockout (LCN2 KO) mice from GemPharmatech. The expression of LCN2 protein was significantly declined in cardiac tissues in LCN2 KO mice (Supplementary Figure 3A).

**FIGURE 2:**
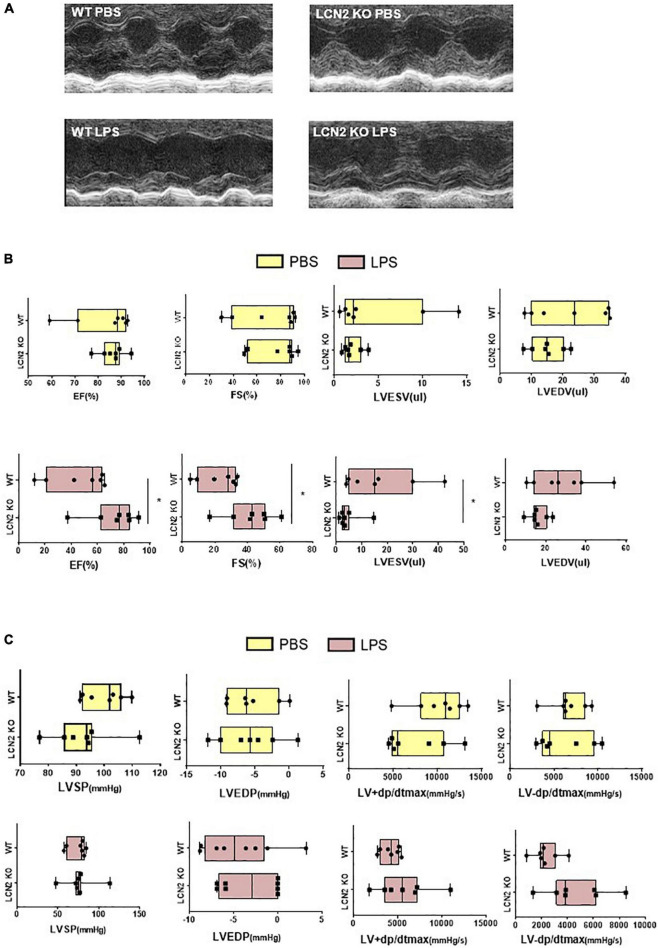
LCN2 deficiency improved cardiac function in LPS-induced SCD mice. **(A,B)** Echocardiography was performed 12 h after intraperitoneal injection of either PBS or LPS (25 mg/kg) to evaluate EF, FS, LVEDV, and LVESV in LCN2 KO mice and their WT littermates (*n* = 7 in each group). **(C)** Invasive hemodynamics was monitored 12 h after intraperitoneal injection with either PBS or LPS (25 mg/kg) in LCN2 KO mice and their WT littermates (*n* = 7 in each group). Measurement data are presented as median with 95% CI. *p < 0.05.

There was no difference of EF, FS, LVEDV, or LVESV at the baseline of LCN2 KO mice and their littermates (Figures 2A,B). But compared to their WT littermates, EF and FS were significantly increased, LVESV was decreased in LCN2 KO mice in response to LPS (Figures 2A,B). Compared to their WT littermates after LPS administration, although there was no significant difference, an increasing tendency of LV-dp/dt max was observed in the LCN2 KO group (*P* = 0.053) (Figure 2C). Taken together, LCN2 deficiency led to the improvement of the cardiac function in LPS-induced mice.

### Lipocalin-2 initiated ferroptosis in cardiomyocytes

**FIGURE 3:**
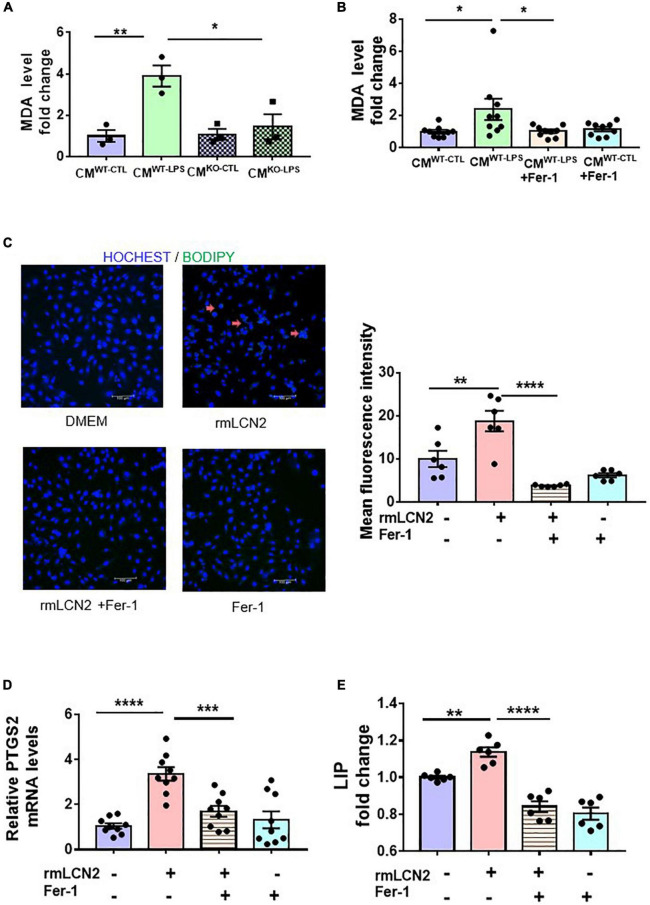
LCN2 initiated ferroptosis in cardiomyocytes. **(A)** Neonatal cardiomyocytes were incubated for 24 h with CMs of the neutrophils from either WT littermates without LPS-preconditioning (CM^WT–CTL^) or with LPS-preconditioning (CM^WT–LPS^), and from LCN2 KO mice without LPS-preconditioning (CM^LCN2–CTL^) or with LPS-preconditioning (CM^LCN2–LPS^) to detect MDA levels (*n* = 3 in each group). **(B)** Neonatal cardiomyocytes were pre-incubated for 2 h with or without Fer-1 (10 μM) prior to incubation with CMs for 24 h to detect MDA levels (*n* = 9 in each group). **(C)** Representative images and fluorescence intensity analysis of H9C2 cells pre-incubated with or without Fer-1 (10 μM) for 2 h prior to incubation with rmLCN2 (1 μg/ml) for 24 h by 581/591C11-BODIPY staining (red arrows indicate the fluorescence staining, *n* = 3 in each group). **(D)** PTGS2 mRNA expressions by qPCR in H9C2 cells pre-incubated with or without Fer-1 (10 μM) for 2 h prior to incubation with rmLCN2 (1 μg/ml) for 24 h (*n* = 9 in each group). **(E)** Labile iron pool (LIP) changes in H9C2 cells pre-incubated with or without Fer-1 (10 μM) for 2 h prior to incubation with rmLCN2 (1 μg/ml) for 24 h (*n* = 6 in each group). Measurement data are presented as mean ± SEM. *p < 0.05, **p < 0.01, ***p < 0.001, ****p < 0.0001.

To explore the potential mechanisms of LCN2 on SCD, lipid peroxidation was measured by the MDA level and BODIPY 581/591 C11 to indicate ferroptosis in cardiomyocytes. CMs of the neutrophils from either WT littermates without LPS preconditioning (CM*^WT^*^–^*^CTL^*) or with LPS preconditioning (CM*^WT^*^–^*^LPS^*), and from LCN2 KO mice without LPS-preconditioning (CM*^LCN^*^2–^*^CTL^*) or with LPS-preconditioning (CM*^LCN^*^2–^*^LPS^*) were harvested individually and incubated with neonatal cardiomyocytes. CM*^WT^*^–^*^LPS^* significantly elevated MDA levels in neonatal cardiomyocytes, which was abrogated by CM*^LCN^*^2–^*^LPS^* (Figure 3A). Consistent results were observed in neonatal cardiomyocytes with CM*^WT^*^–^*^LPS^* by Fer-1, a specific ferroptosis inhibitor (Figure 3B).

Next, exogenous recombinant LCN2 (rmLCN2) was adopted to incubate with H9C2 cells for 24 h. BODIPY 581/591 C11 staining and mean fluorescence intensity demonstrated that exogenous rmLCN2 enhanced lipid peroxidation in H9C2 cells (Figure 3C). Furthermore, exogenous rmLCN2 incubation significantly promoted prostaglandin E synthase 2 (PTGS2) mRNA expression (Figure 3D) and LIP levels (Figure 3E), which were diminished by Fer-1 (Figures 3C–E). However, exogenous rmLCN2 incubation did not alter glutathione peroxidase 4 (GPx4) protein levels by Western blot (Supplementary Figure 4A). Collectively, our findings suggested that LCN2 triggered ferroptosis in cardiomyocytes, independent of GPx4.

### Lipocalin-2 induced ferroptosis *via* increasing labile iron pool in H9C2

**FIGURE 4:**
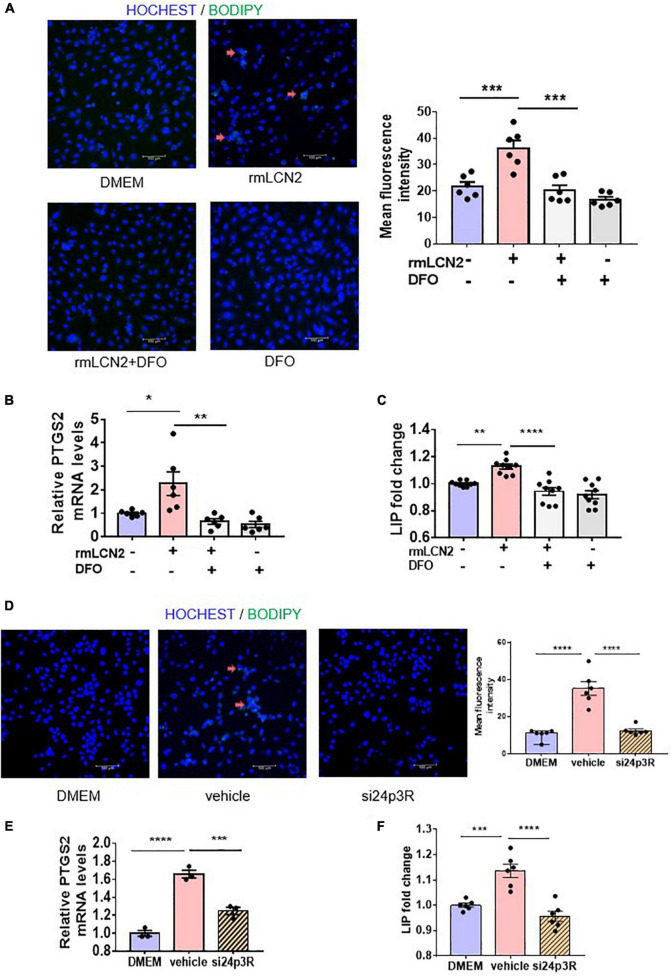
LCN2 induced ferroptosis *via* increasing LIP in H9C2 cells. **(A)** Representative images and fluorescence intensity analysis of H9C2 cells pre-incubated with or without DFO (25 μM) for 2 h prior to incubation with rmLCN2 (1 μg/ml) for 24 h by 581/591C11-BODIPY staining (red arrows indicate the fluorescence staining, *n* = 3 in each group). **(B)** PTGS2 mRNA expressions by qPCR in H9C2 cells pre-incubated with or without DFO (25 μM) for 2 h prior to incubation with rmLCN2 (1 μg/ml) for 24 h (*n* = 6 in each group). **(C)** LIP changes in H9C2 cells pre-incubated with or without DFO (25 μM) for 2 h prior to incubation with rmLCN2 (1 μg/ml) for 24 h (*n* = 9 in each group). **(D)** Representative images and fluorescence intensity analysis of H9C2 cells pre-incubated with or without 24p3R siRNA for 48 h prior to incubation with rmLCN2 (1 μg/ml) for 24 h by 581/591C11-BODIPY staining (red arrows indicate fluorescence staining, *n* = 3 in each group). **(E)** PTGS2 mRNA expressions by qPCR in H9C2 cells pre-incubated with or without 24p3R siRNA for 48 h prior to incubation with rmLCN2 (1 μg/ml) for 24 h *n* = 3 in each group). **(F)** LIP changes in H9C2 cells pre-incubated with or without 24p3R siRNA for 48 h prior to incubation with rmLCN2 (1 μg/ml) for 24 h (*n* = 6 in each group). *p < 0.05, **p < 0.01, ***p < 0.001, ****p < 0.0001.

To interpret the intracellular modulation of iron dynamic equilibrium by LCN2, H9C2 cells were incubated with or without deferoxamine (DFO), an iron chelator, while adapted with exogenous rmLCN2. DFO incubation obviously attenuated fluorescence staining and mean fluorescence intensity by BODIPY 581/591 C11 in H9C2 cells (Figure 4A), as well as PTGS2 mRNA expression and LIP levels (Figures 4B,C).

The membrane receptor, 24p3R, was expressed in H9C2 cells (34). Compared to the non-specific siRNA negative control (vehicle), 24p3R-siRNA (si24p3R) incubation markedly attenuated fluorescence staining and mean fluorescence intensity by BODIPY 581/591 C11 in H9C2 cells (Figure 4D), as well as PTGS2 mRNA expression and LIP levels (Figures 4E,F). These results indicated that rmLCN2 induced H9C2 ferroptosis by increasing LIP *via* 24p3R.

### Septic cardiac dysfunction mice have increased ferroptosis

**FIGURE 5:**
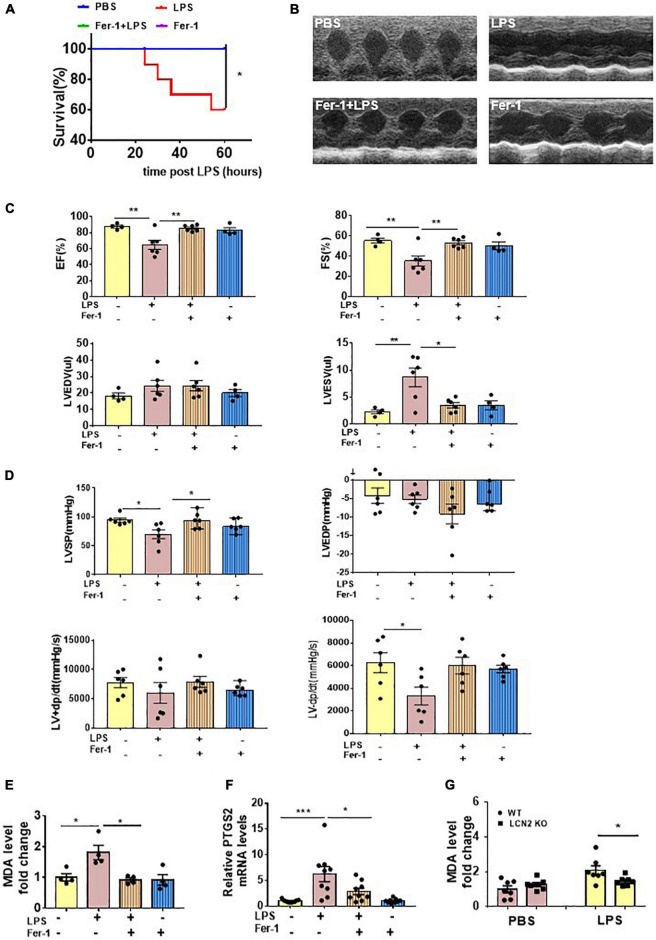
Ferroptosis presented in LPS-induced SCD mice. **(A)** Survival curve of WT mice intraperitoneally injected with either PBS or Fer-1 (1 mg/kg) at 24 h, 2 h before LPS (25 mg/kg, *n* = 10 in each group) administration. **(B,C)** Echocardiography was performed in WT mice intraperitoneally injected with either PBS or Fer-1 (1 mg/kg) at 24 h, 2 h followed by LPS (25 mg/kg) or PBS administration for 12 h (*n* = 4, 6, 6, 4, respectively). **(D)** Invasive hemodynamics was monitored in WT mice intraperitoneally injected with either PBS or Fer-1 (1 mg/kg) at 24 h, 2 h followed by LPS (25 mg/kg) or PBS administration for 12 h (*n* = 6 in each group). **(E)** Cardiac MDA levels were detected in WT mice intraperitoneally injected with either PBS or Fer-1 (1 mg/kg) at 24 h, 2 h followed by LPS (25 mg/kg) or PBS administration for 12 h (*n* = 4 in each group). **(F)** Cardiac PTGS2 mRNA expressions were determined by qPCR in WT mice intraperitoneally injected with either PBS or Fer-1 (1 mg/kg) at 24 h, 2 h followed by LPS (25 mg/kg) or PBS administration for 12 h (*n* = 9 in each group). **(G)** Cardiac MDA levels were detected in LCN2 KO mice and their WT littermates 12 h after intraperitoneal injection of either PBS or LPS (25 mg/kg) (*n* = 7 in each group). *p < 0.05, **p < 0.01, ***p < 0.001.

To evaluate the effect of ferroptosis inhibition on LPS-induced SCD, 6 to 8-week-old male C57Bl/6 mice were intraperitoneally injected with or without Fer-1 (1 mg/kg) 24 and 2 h prior to LPS administration. At termination, 60% mice eventually survived in the LPS-induced group, whereas no death occurred in the Fer-1 pre-treatment group (Figure 5A). Compared to the LPS group, EF and FS were significantly increased, and LVESV was significantly decreased in mice administrated with Fer-1 assessed by echocardiography (Figures 5B,C). The Fer-1-pretreated mice displayed an increase in LVSP, and a tendency of increased LV-dp/dt max was also been observed, although there was no significant difference (*P* = 0.0651) (Figure 5D) compared to SCD mice.

LPS induced elevations of cardiac MDA levels and PTGS2 mRNA levels in the WT mice, which were abrogated by Fer-1 administration (Figures 5E,F). The LCN2 protein level was upregulated in LPS-induced SCD mice, which was not affected by Fer-1 administration (Supplementary Figure 5A). Furthermore, cardiac MDA levels were declined in LCN2 KO mice compared to their WT littermates in response to LPS (Figure 5G).

We also measured other biomarkers of ferroptosis in heart tissues of SCD mice. However, no changes had been observed in ferroptosis suppressor protein 1 (FSP1) and GPx4 protein levels (Supplementary Figure 6A) and mRNA levels of acyl-CoA synthetase long-chain family member 4 (ACSL4), 12-lipoxygenases (ALOX12), and 15-lipoxygenases (ALOX15) (Supplementary Figure 6B). qPCR demonstrated that mRNA levels of SLC7A11 were significantly increased in heart tissues of SCD mice, which was not abrogated by Fer-1 administration (Supplementary Figure 6C). Thus, ferroptosis is involved in LPS-induced SCD, and it can be inhibited by Fer-1.

We further analyzed neutrophil infiltration in the WT and LCN2 KO mice. Inconsistent to the previous studies (35, 36), no difference of neutrophil infiltration (as CD45 + F4/80-Ly6G + cells by flow cytometry) was observed in the BM, blood, and heart tissue after intraperitoneal injection with either PBS or LPS (Supplementary Figures 7A,B). In addition, the mRNA expressions of IL-6, IL-1β, and TNFα in heart tissues by qPCR were not significant different between the WT mice and LCN2 KO mice (Supplementary Figure 7C). These data suggested that cardiac dysfunction and ferroptosis could be attenuated by LCN2 deficiency, independent of neutrophil recruitment.

## Discussion

In the present study, we established an LPS-induced SCD murine model and generated LCN2 KO mice to investigate the role and the mechanisms of LCN2 in the development of SCD. Herein, LCN2 was highly expressed in cardiac tissue and accounted for heart failure in LPS-induced SCD mice, which dominantly originated from peripheral neutrophils. *In vitro* experiments showed that LCN2 was involved in myocardial ferroptosis by increasing intracellular LIP *via* 24p3R. LCN2 deficiency attenuated cardiac ferroptosis and improved cardiac function in SCD mice, which might be a potential therapeutic target for SCD patients.

LCN2, a member of neutrophil granule markers, is stored in the specific granules and can be carried to the local infection area (37). Regarded as a biomarker of infected diseases, LCN2 offers protection against *E. coli*-induced septicemia (38), pneumonia (39), and urinary tract infection (40). According to a previous study (3), we explored the LCN2 expression in cardiomyocytes, cardiac fibroblasts, neutrophils, and macrophages in response to LPS. Cell experiments showed that LCN2 was much more expressed in neutrophils than in other cells. Accordingly, based on the findings that neutrophils were dramatically infiltrated in cardiac tissue and LCN2 expression was upregulated in peripheral neutrophils of the LPS-induced SCD mice, we found that LCN2 was mainly derived from peripheral neutrophils.

LCN2 is widely reported to be involved in acute kidney injury (41, 42) and chronic kidney disease (43) and is a biomarker of hepatic ischemia reperfusion injury severity stages (44). As reported previously, LCN2 was widely distributed in the liver, lung, kidney, and other organs as an acute inflammatory protein in sepsis mice (45). Here, we reported that LCN2 was accumulated in LPS-induced cardiac tissue and contributed to heart failure, whereas LCN2 deficiency improved cardiac function. Future studies need to be designed to evaluate the function of LCN2 in other types of heart failure.

Ferroptosis is defined in 2012 as an iron-dependent regulated form of cell death, caused by intracellular accumulation of lipid-based reactive oxygen species (ROS) (46, 47). Relevant conditions underlying cardiac redox imbalance include iron overload associated with ROS production *via* the Fenton reaction and the magnitude of the LIP achieved (48, 49). For instance, it has demonstrated that free iron released on heme degradation is necessary and sufficient to induce cardiac injury, both in doxorubicin-induced cardiomyopathy and ischemia/reperfusion (I/R) injury (21, 50). Consistent with the previous result (16, 25), our study indicated that ferroptosis played a critical role in LPS-induced cardiac injury. In previous experimental studies, LCN2 deficiency improved the cardiac function by attenuating cardiac hypertrophy in human hypertrophic cardiomyopathy (51). Otherwise, LCN2 can exacerbate cardiac dysfunction by suppressing the beneficial cardiac autophagic response and accelerate cardiac apoptosis in myocardial infarction (52). As an iron transporter, LCN2 can deliver siderophore-bound iron into cells and increase cytoplasm LIP (53), which is supported by our study that exogenous rmLCN2 increased intracellular LIP in H9C2 cells and led to ferroptosis. Also, LCN2 deficiency decreased ferroptosis in LPS-induced mice.

**FIGURE 6:**
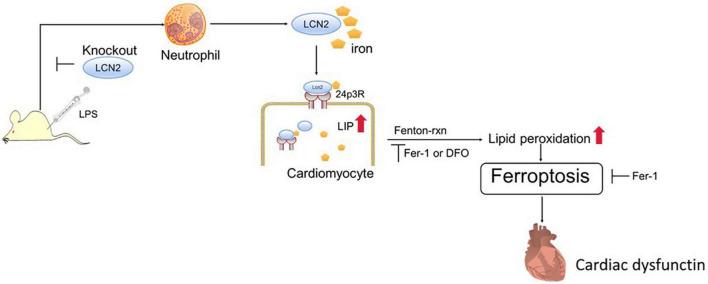
LCN2 and its potential mechanism on SCD. Neutrophil-derived LCN2 initiates intracellular LIP elevation *via* 24p3R, triggers ferroptosis, and eventually contributes to cardiac dysfunction.

To verify ferroptosis in LPS-induced SCD, ferroptosis-specific inhibitor (Fer-1) and iron chelator (DFO) were adopted in this study. As expected, both agents attenuated exogenous LCN2-induced ferroptosis in H9C2 cells. 24p3R, a dominant membrane receptor, was expressed in H9C2 cells. The blockade of 24p3R resulted in ferroptosis alleviation in H9C2 cells. The potential mechanism of LCN2 on SCD may be explained as the follows (Figure 6). LPS induced recruitment of peripheral neutrophils to cardiac tissues, followed by the release of LCN2. Neutrophil-derived LCN2 carried iron into cardiomyocytes *via* 24p3R and initiated intracellular LIP elevation, thereby leading to ferroptosis independent of GPx4, which could be reversed by both Fer-1 and DFO. It is indicated that targeted inhibition of LCN2 may act as a promising approach for the treatment of septic cardiomyopathy.

In summary, our study elucidates that neutrophil-derived LCN2 initiates intracellular LIP elevation *via* 24p3R and triggers ferroptosis in cardiomyocytes. SCD and ferroptosis could be attenuated by LCN2 deficiency, which implicated that LCN2 may act as a promising target for the treatment of septic cardiomyopathy.

## Data availability statement

The original contributions presented in this study are included in the article/Supplementary material, further inquiries can be directed to the corresponding author/s.

## Ethics statement

The animal study was reviewed and approved by Second Affiliated Hospital, Zhejiang University School of Medicine.

## Author contributions

YH conducted the experiments, analyzed the results, and wrote the manuscript draft. CX, NZ, and YY participated in specific experiments and analyzed the results. YH and FY provided conceptual advice and revised the draft. XX and JW designed the study and modified the manuscript draft. All authors read and approved the final manuscript.

## Abbreviations

LCN2: lipocalin-2
LIP: labile iron pool
MDA: malonaldehyde
PTGS2: prostaglandin E synthase 2
rmLCN2: recombinant LCN2
GPx4: glutathione peroxidase 4
FSP1: ferroptosis suppressor protein 1
ACSL4: acyl-CoA synthetase long-chain family member 4
ALOX12: 12-lipoxygenases
ALOX15: 15-lipoxygenases
Fer-1: ferrostatin-1
DFO: deferoxamine
SCD: septic cardiac dysfunction.
