# Supplementary material for: Lipocalin-2 in neutrophils induces ferroptosis in septic cardiac dysfunction via increasing labile iron pool of cardiomyocytes
Source: Front Cardiovasc Med. 2022 Aug 4;9:922534. doi: 10.3389/fcvm.2022.922534 (PMC9386130; doi:10.3389/fcvm.2022.922534)
Supplement: Supplementary file 1 [file Presentation_1.pdf]

A

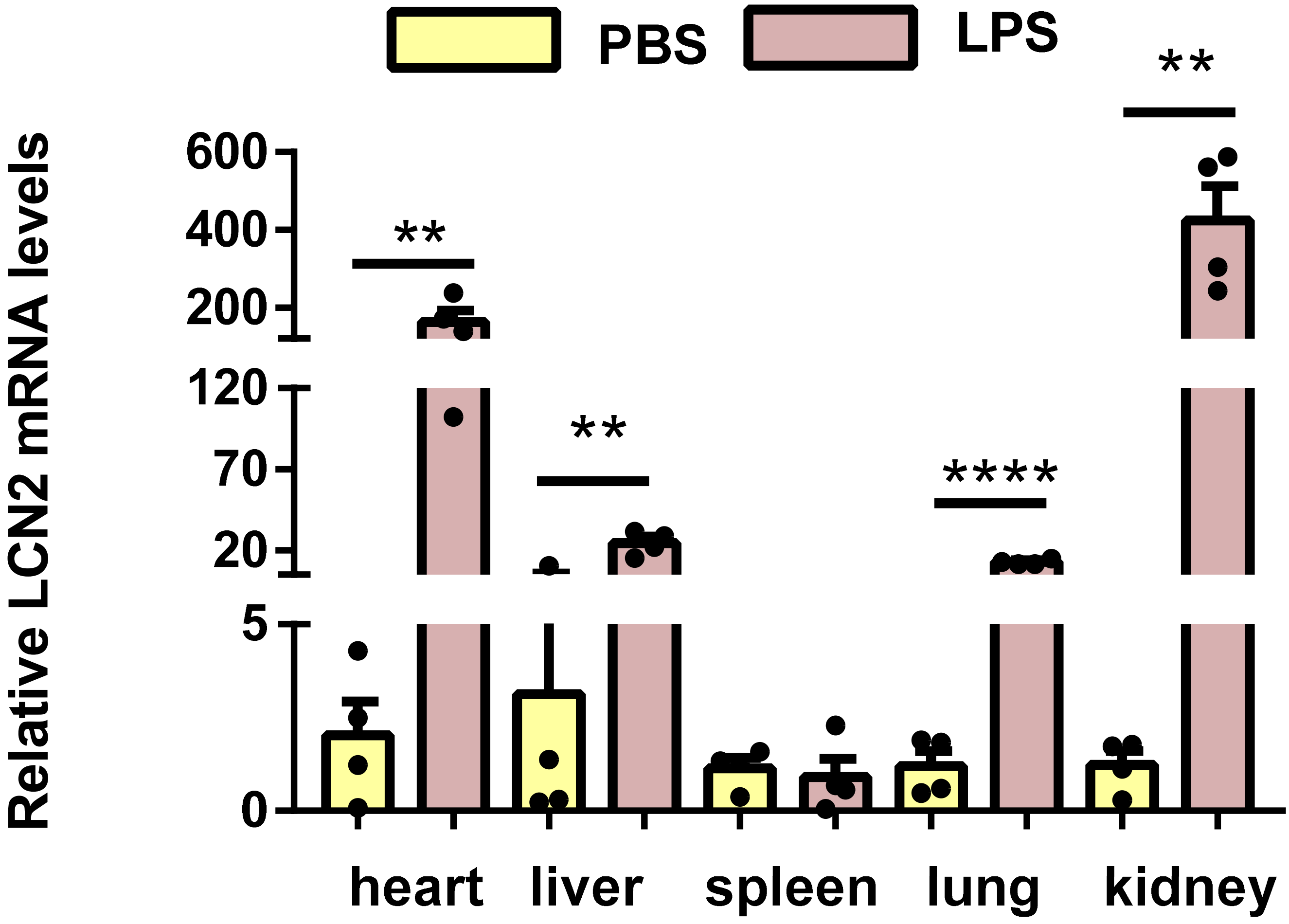

Fig S1. LCN2 expression of tissues increased in LPS induced mice.(A) Quantitative PCR analysis of LCN2 mRNA expressions in the tissues 12 h after intraperitoneal injection with either PBS or LPS in WT mice(n=4 in each group. Measurement data are presented as mean ±SEM.**p<0.01, ****p<0.0001.

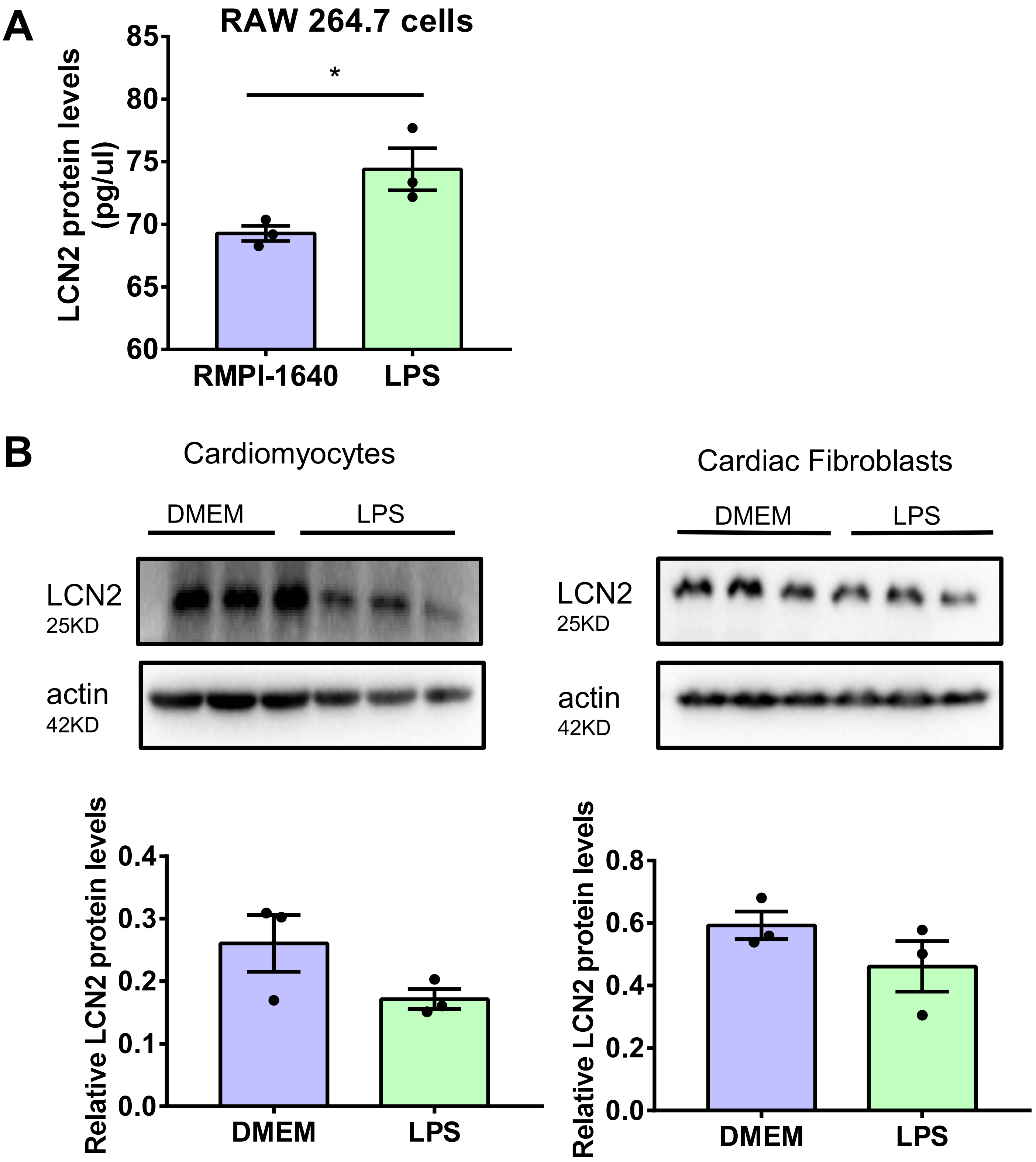

Fig S2. **LCN2 expression in RAW 264.7 cells ,cardiomyocytes and fibroblasts incubated with LPS.(A)**ELISA detection of the LCN2 in the RAW 264.7 cells after 12 h incubation with either RMPI-1640 or LPS (100ng/ml, n=3 in each group). **(B)** Western blot detection and quantitative analysis of the LCN2 protein levels in the cardiomyocytes and cardiac fibroblasts after 24 h incubation with either DMEM or LPS (1ug/ml, n=3 in each group). Measurement data are presented as mean ±SEM,*p<0.05.

A

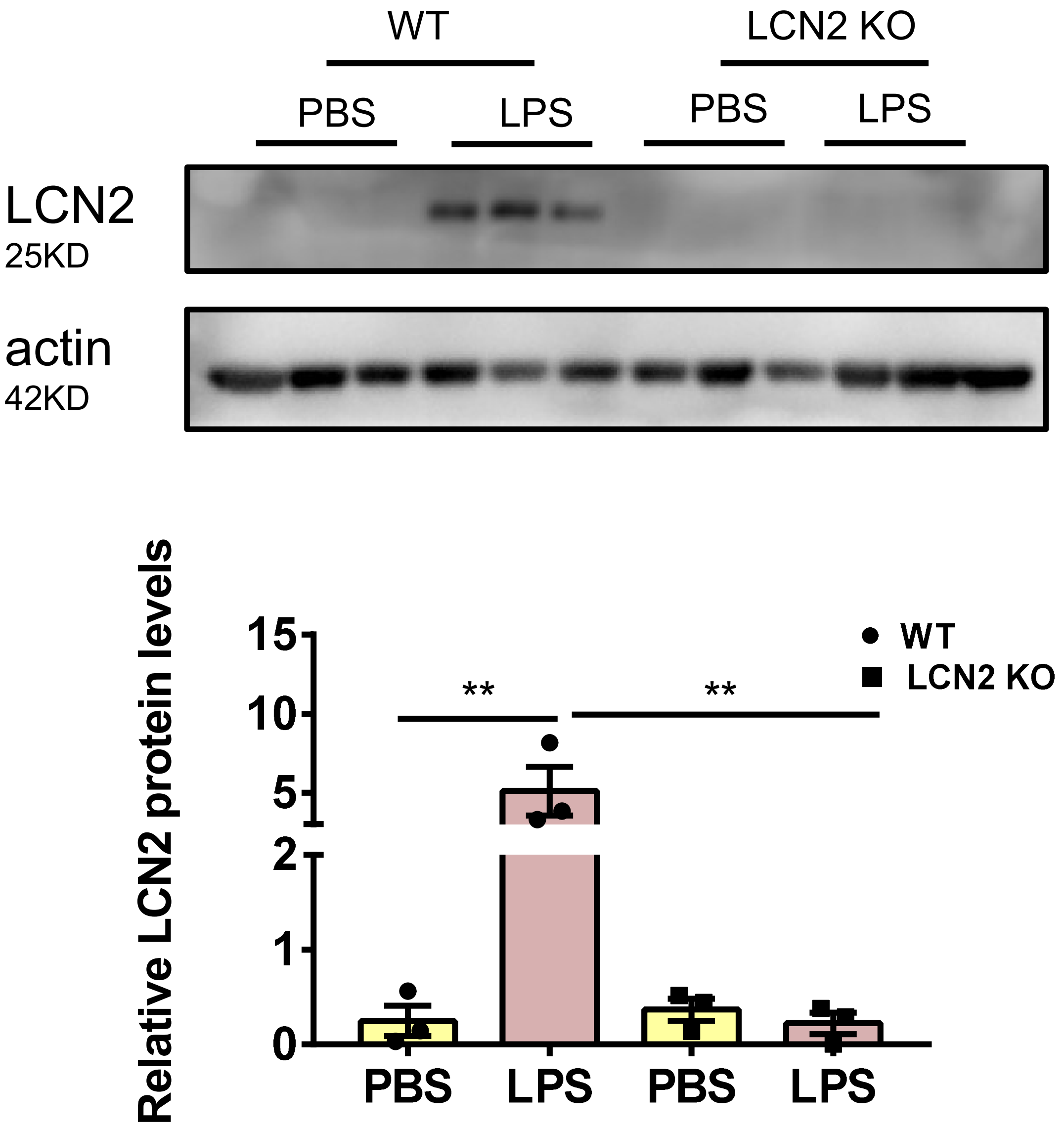

Fig S3.LCN2 expression decreased in LCN2 KO mice.

(A) Western blot detection and quantitative analysis of the LCN2 protein levels of LCN2 in heart tissues 12 h post intraperitoneal injection with either PBS or LPS in LCN2 KO mice and their WT littermates (n=3 in each group). Measurement data are presented as mean ±SEM,**p<0.01.

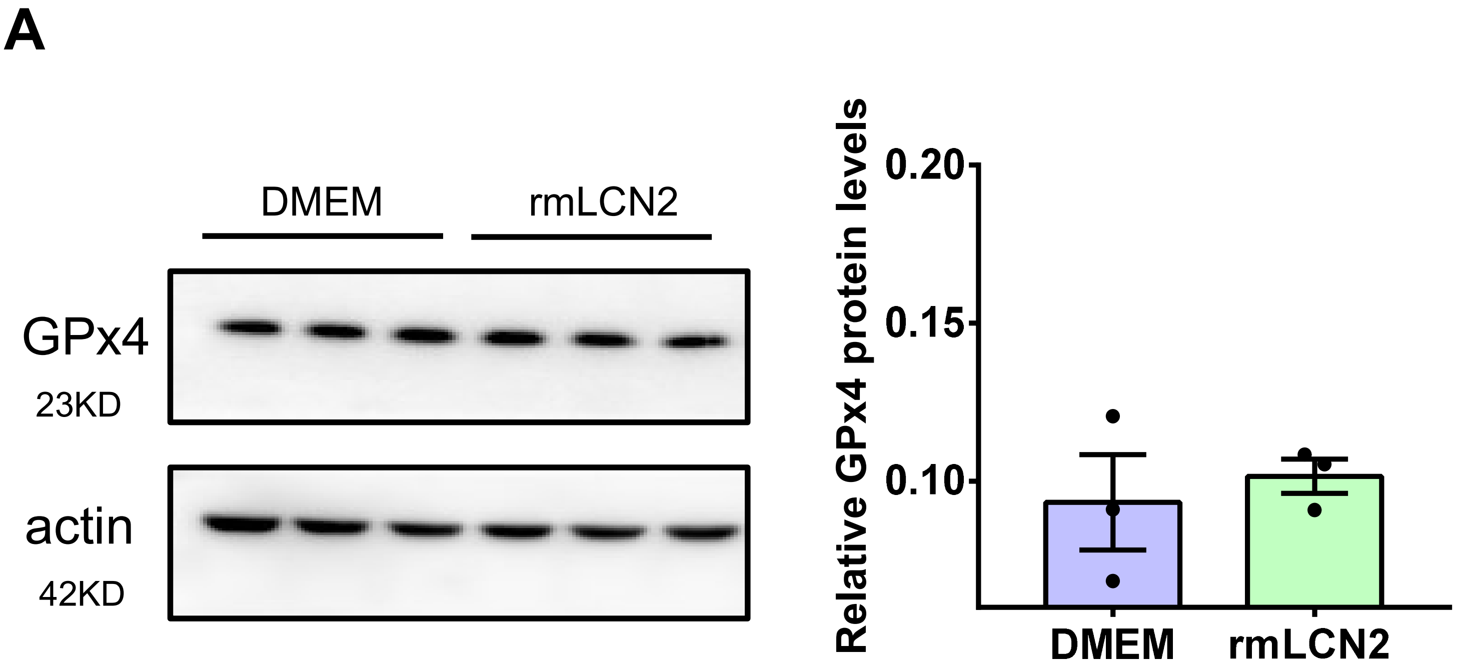

Fig S4. Exogenous LCN2 didn’t change GPx4 protein levels of H9C2.(A) Western blot detection and quantitative analysis of the GPx4 protein levels of H9C2 incubated with either DMEM or rmLCN2 (1μg/ml) for 24 h(n=3 in each group). Measurement data are presented as mean ±SEM.

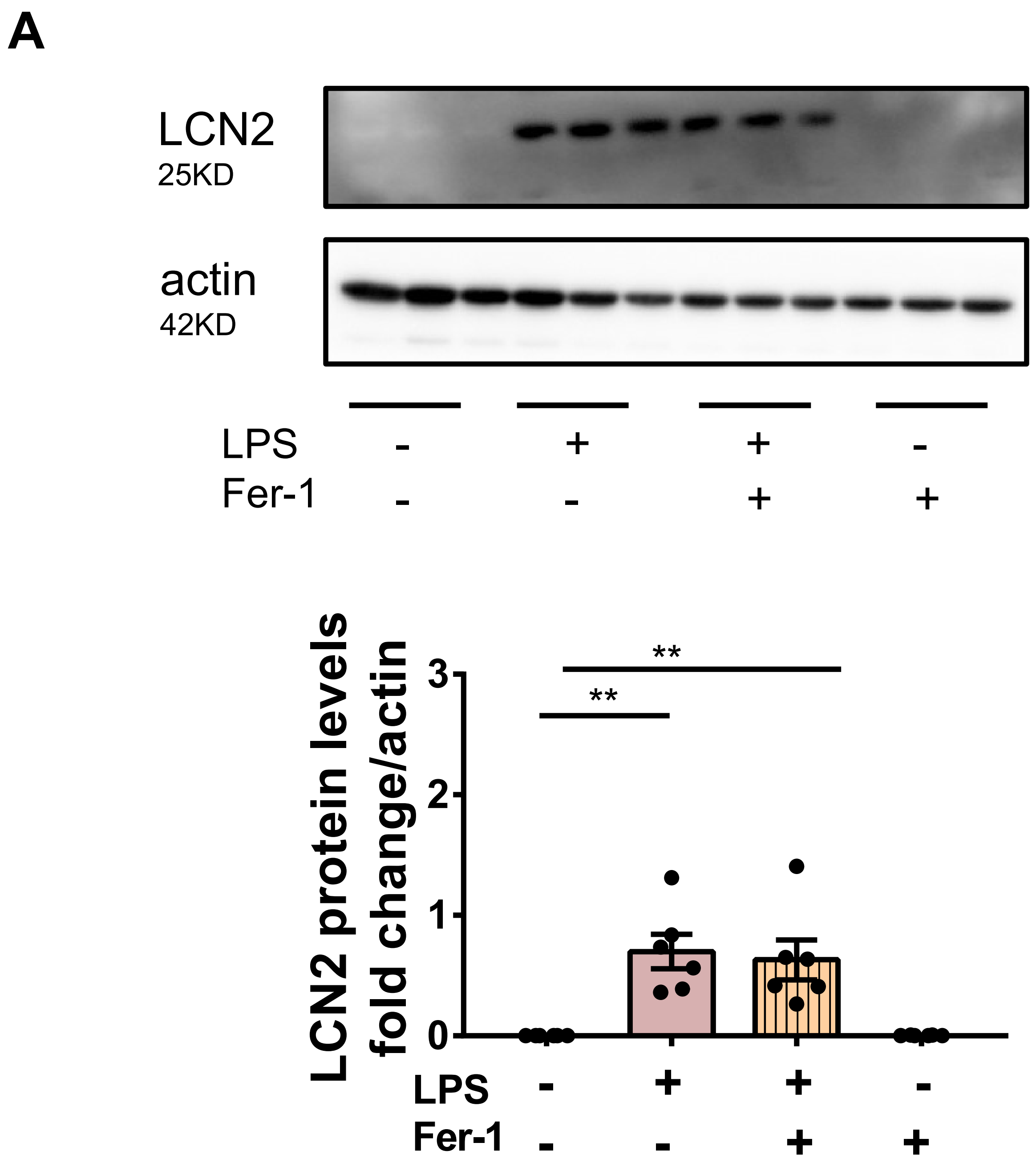

Fig S5.Fer-1 had no effect of LCN2 expression in heart tissues of SCD mice.(A) Western blot detection and quantitative analysis of the LCN2 protein levels in heart tissues of WT mice intraperitoneally injected either PBS or Fer-1(1mg/kg) at 24 h, 2 h before LPS (25mg/kg) administration(n=6 in each group). Measurement data are presented as mean ±SEM.**p<0.01.

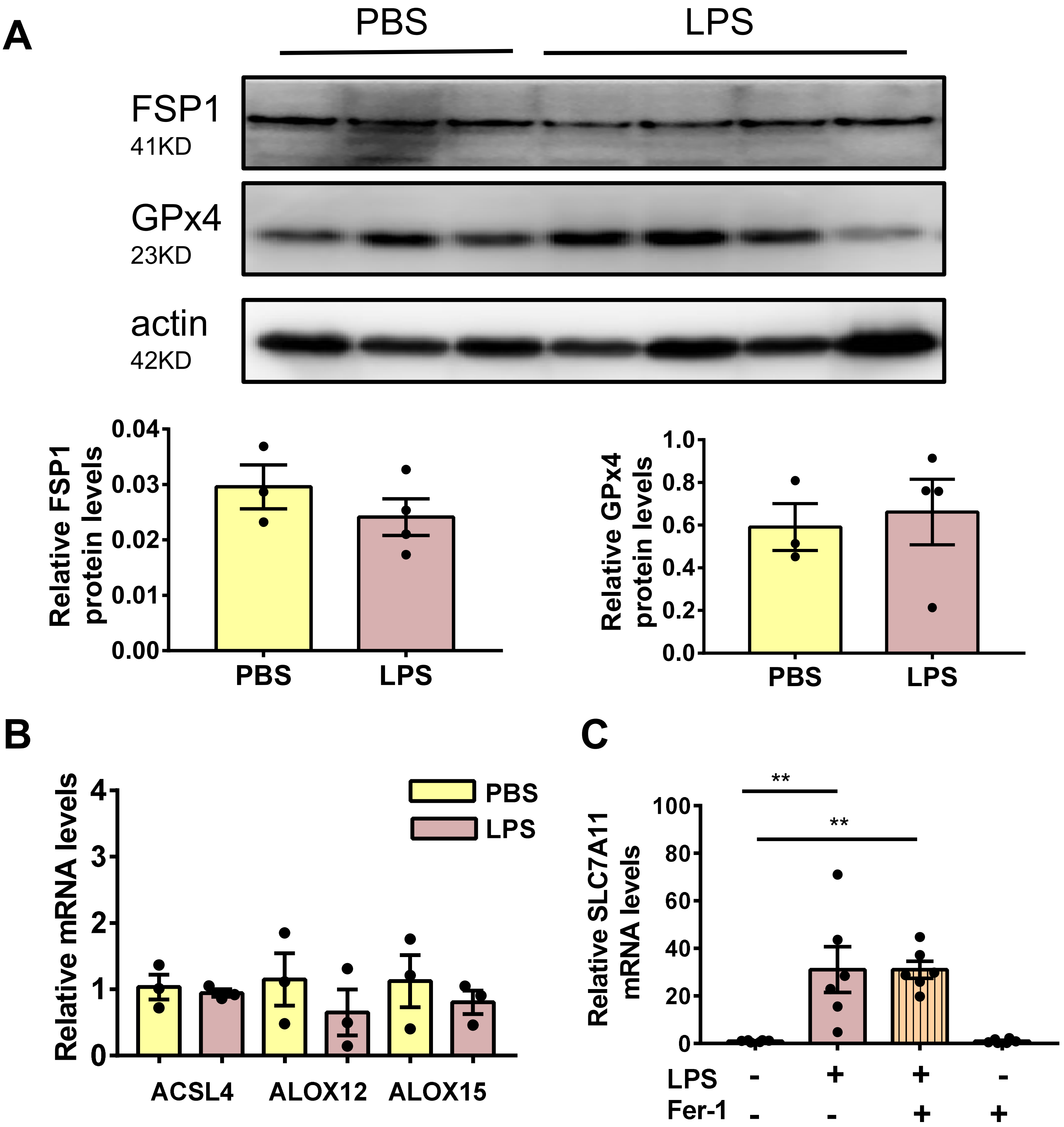

Fig S6. Ferroptosis markers in SCD mice. (A) Western blot detection and quantitative analysis of the FSP1 and GPx4 protein levels in the heart tissues 12h after intraperitoneal injection with either PBS(n=3) or LPS(25mg/kg, n=4) in WT mice. **(B)** Quantitative PCR analysis of mRNA levels of ACSL4 ,ALOX12,ALOX15 in the heart tissues 12h after intraperitoneal injection with either PBS or LPS(25mg/kg) in WT mice(n=3 in each group). **(C)** Quantitative PCR analysis of mRNA levels of SLC7A11 in the heart tissues 12h of WT mice intraperitoneally injected with either PBS or Fer-1(1mg/kg) at 24 h, 2 h before LPS (25mg/kg) administration(n=6 in each group). Measurement data are presented as mean ±SEM.**p<0.01.

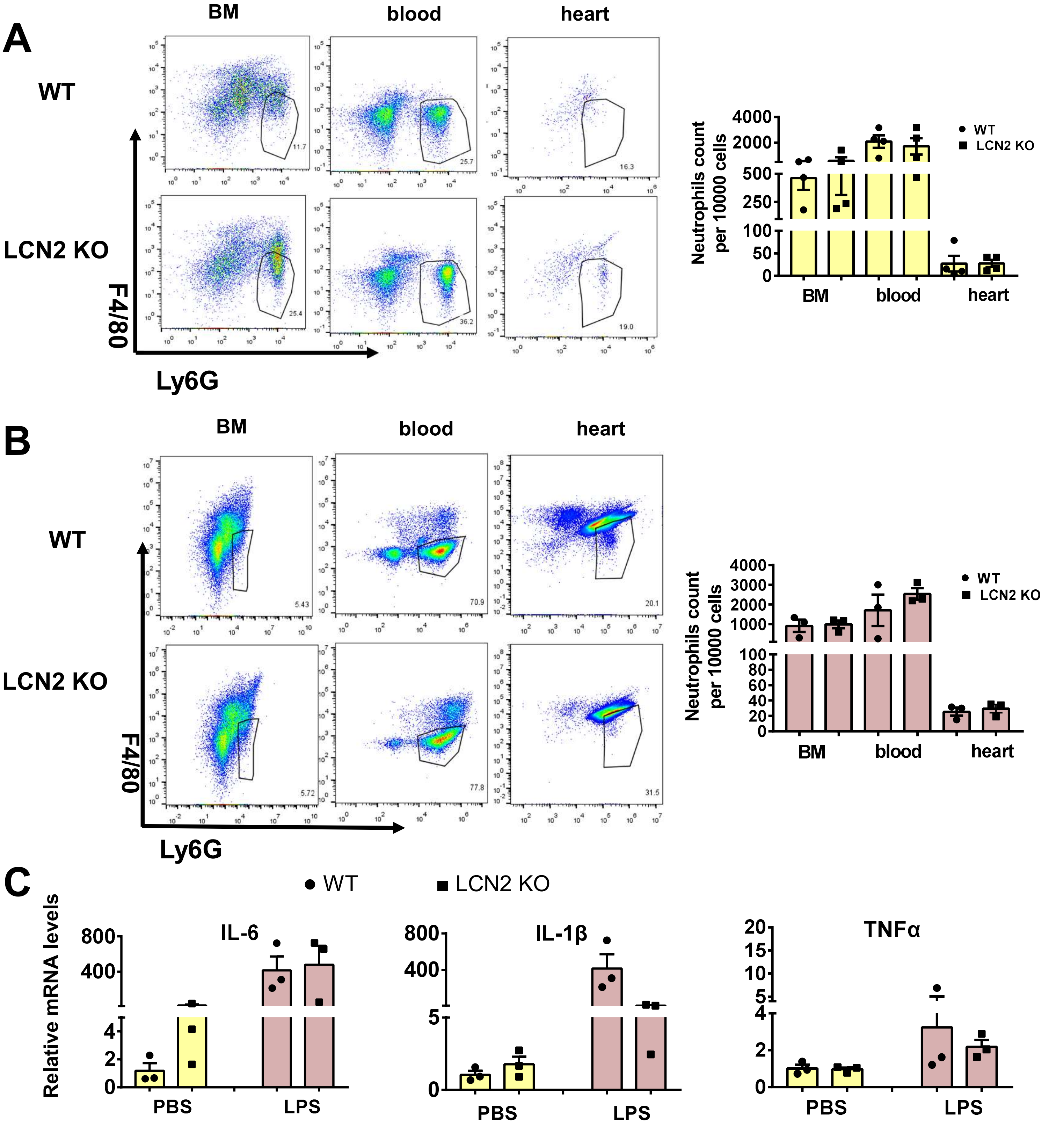

Fig S7.Neutrophils infiltration , proinflammatory cytokine in LCN2 KO and WT mice. (A) Flow cytometric analysis of the number of CD45+ F4/80-Ly6G+ cells among the bone marrow(BM),blood and isolated heart cells 12 h post intraperitoneal injection with PBS in LCN2 KO mice and their WT littermates (n=4 in each group). **(B)** Flow cytometric analysis of the number of CD45+F4/80- Ly6G+ cells among the bone marrow(BM),blood and isolated heart cells 12 h post intraperitoneal injection with LPS(25mg/kg) in LCN2 KO mice and their WT littermates (n=3 in each group). **(C)** Quantitative PCR analysis of mRNA levels of Il-6,IL-1β,TNF α in mice 12 h post intraperitoneal injection with either PBS or LPS(25mg/kg) in LCN2 KO mice and their WT littermates (n=3 in each group).Measurement data are presented as mean ±SEM.
